# Effect of Jute Fibres on the Process of MICP and Properties of Biocemented Sand

**DOI:** 10.3390/ma13235429

**Published:** 2020-11-28

**Authors:** Christine Ann Spencer, Leon van Paassen, Henrik Sass

**Affiliations:** 1 School of Engineering, Cardiff University, Cardiff CF24 3AA, UK; 2 Center for Bio-Mediated and Bio-Inspired Geotechnics (CBBG), Arizona State University, Tempe, AZ 85287-3005, USA; leon.vanpaassen@asu.edu; 3 School of Earth and Ocean Sciences, Cardiff University, Cardiff CF10 3AT, UK; sassh@cardiff.ac.uk

**Keywords:** biocementation, MICP, jute fibres, unconfined compressive strength, urea hydrolysis, sustainable geotechnics, self-healing

## Abstract

There has been increasing interest, in the past decade, in bio-mediated approaches to soil improvement for geotechnical applications. Microbially induced calcium carbonate precipitation (MICP) has been investigated as a potentially sustainable method for the strengthening and stabilisation of soil structures. This paper presents the results of a study on the effect of jute fibres on both the MICP process and properties of biocemented sand. Ureolytic *Sporosarcina pasteurii* has been used to produce biocemented soil columns via MICP in the laboratory. Results showed that columns containing 0.75% (by weight of sand) untreated jute fibres had unconfined compressive strengths approximately six times greater on average compared to biocemented sand columns without jute fibres. Furthermore, efficiency of chemical conversion was found to be higher in columns containing jute fibres, as measured using ion chromatography. Columns containing jute had calcimeter measured CaCO_3_ contents at least three times those containing sand only. The results showed that incorporation of jute fibres into the biocemented sand material had a beneficial effect, resulting in stimulation of bacterial activity, thus sustaining the MICP process during the twelve-day treatment process. This study also explores the potential of jute fibres in self-healing MICP systems.

## 1. Introduction

Growing interest in sustainable methods of soil improvement has led to increased interest and research in the application of microbially induced calcium carbonate precipitation (MICP). MICP is a biogeochemical process which can be used to improve the mechanical properties of loose, saturated sand, by increasing its strength and stiffness and reducing its tendency to dilate [1]. MICP may be used as an alternative to the traditional Portland cement-based method of soil cementation [2]. MICP may occur through a variety of metabolic pathways, including photosynthesis, ureolysis, ammonification, denitrification and methane oxidation [3], in addition to sulphate reduction and iron reduction [4]. Ureolysis was selected for this study. This process is dependent upon suitable bacteria and cementation media. Ureolysis increases the alkalinity of fluid in soil pore spaces as a result of the degradation of urea to carbonate and ammonium (Equation (1)), and induces calcium carbonate precipitation (Equation (2)) [5].

(1) $$\mathrm{CO}{\left({\mathrm{NH}}_{2}\right)}_{2}\;+\;2H_{2}O\to 2{\mathrm{NH}}_{4}^{+}+{\mathrm{CO}}_{3}^{2-}$$

(2) $${\mathrm{Ca}}^{2+}+{\;\mathrm{CO}}_{3}^{2-}\to {\;\mathrm{CaCO}}_{3\left(S\right)}$$

Precipitation of calcium carbonate (CaCO_3_) leads to pore-filling, inter-particle binding and particle roughening; resulting in improved soil strength and stiffness and also reduced permeability [6].

Urease positive *Sporosarcina pasteurii* (formerly *Bacillus pasteurii*) has commonly been used for studies on cementation of granular soil via MICP [3,5,7,8,9]. *S. pasteurii* is tolerant to a wide pH range. Stocks-fischer et al. 8 reported urease activity between pH 6 and 10, with the optimum environment being slightly alkaline. *Sporosarcina ureae* has also been shown to promote MICP via ureolysis, as demonstrated in preliminary work by Spencer and Sass [10], and by Botusharova [11]; however, the process is much slower when compared to using *S. pasteurii*. Both *S. pasteurii* and *S. ureae* will express urease, regardless of ammonia compound concentrations [12] and are spore-forming.

Recent studies have explored the enhancement of biocemented soils with additional materials such as fibres, these have focussed on mostly synthetic fibres. Fibres utilised to improve strength characteristics have included polyvinyl alcohol (PVA) [13], polypropylene (Fibermesh 150) [14], basalt fibres [15] and carbon fibres [16]. Gao et al. [17] used polypropylene fibres in an MICP based surficial treatment of sand for seepage control. Of these examples only basalt fibres are a natural material, these are however a finite resource.

This study investigates the use of jute fibres, as an innovative approach to enhance the properties of MICP treated sand using sustainable natural fibres. The use of fibres to reinforce soil is an established technique, first proposed by Vidal [18] in 1969. Jute fibres are widely used in building materials, textiles and packaging. Jute is the most common natural fibre cultivated in the world, it is biodegradable and has good tensile strength [19]. Natural fibres, such as jute, are affordable and recyclable. For structural applications, fibres may be premixed with soil during construction or incorporated *in situ* using deep mixing techniques. Fibres can be mixed with soil to construct embankment dams and other water-retaining structures to improve resistance to piping erosion, with fibre content determined by suitable piping tests [20].

This current study, which combines jute fibres and MICP, has been completed as part of an investigation into the feasibility of the use of jute and other absorbent materials to enable autonomous self-healing of biocemented sand via MICP [10]. Self-healing, in the context of construction materials, can be defined as, ‘the partial or total recovery of at least one property of a material’ [21]. As a further enhancement of a biocemented sand material self-healing is of interest to improve the sustainability of biocemented soil structures and reduce future maintenance and repair costs. Under loading (shear, tension, compression) the calcium carbonate binding between the silica sand particles may fail. The likely failure mechanism being a fracture within the precipitated calcium carbonate or between the precipitate and the silica sand particles [22].

Previous studies by Montoya and Dejong [1] and Botusharova [11] demonstrated that, by injecting the nutrients and precursor chemicals (cementation medium) required for the MICP process into degraded biocemented sand, strength regain could be achieved. To enable biocemented sand to self-heal, provided viable spores of *S. pasteurii* are present, would require a store of this cementation medium within the biocemented sand matrix. The development of a biocemented sand material that can autonomously self-heal is novel and has been inspired by previous studies on self-healing of cementitious materials using immobilisation, as summarised by Spencer and Sass [10]. Having identified jute as a potentially suitable carrier material for immobilisation during preliminary studies, further investigation was required on the effectiveness of jute to store cementation medium and release this to achieve self-healing.

Laboratory experiments have been undertaken to (i) determine the effect of jute fibres on the process of MICP, (ii) quantify effect of jute fibres on strength properties when incorporated into a biocemented sand material, (iii) further investigate potential for use of jute fibres in self-healing geotechnical systems.

## 2. Materials and Methods

### 2.1. Soil and Testing of Properties

A fine F60 foundry sand (U.S. Silica, Ottawa, IL, USA) was selected for this study since the optimum grain size for MICP is 0.05 mm to 0.4 mm [23]. Particle size distribution tests were conducted in accordance with ASTM D6913/D6913M–17 [24], to verify the provided product data, in addition to Proctor compaction tests, in accordance with ASTM D1557-12e1 [25], to establish target density for the sand columns. Properties as otherwise reported by U.S. Silica Company (Ottawa, IL, USA) are as per Table 1.

Prior to use, the sand was autoclaved at 120 °C for 20 min and oven dried at 105 °C for sterilisation purposes. The F60 sand was found to have negligible calcium carbonate content when tested using a calcimeter, and therefore no further treatment of the sand was required.

### 2.2. Fibre Preparation

**Figure 1:**
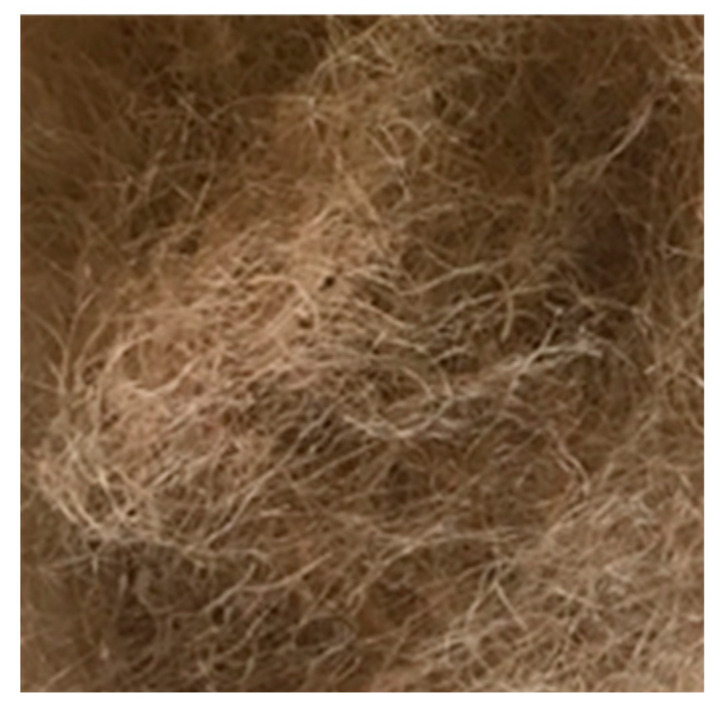
Jute fibres (not to scale).

Natural jute fibres (Sunrise Agriculture, Ajmer, Rajasthan, India) were used for this study. The fibres were initially of variable length, as shown in Figure 1 prior to processing. Clumps of fibres were gently brushed and then hand cut to approximately 6 mm. The length of fibres (*n* = 20) averaged 5.88 mm ± 1.60. A scanning electron microscope (SEM; SNE-4500M Plus Tabletop, SEC, Suwon-si, Korea) was used in this study to measure the diameter of the fibres following the production of the biocemented sand columns.

Prior to use, the jute fibres were washed thoroughly using a sieve and deionised water, followed by autoclaving at 121 °C for 20 min. The fibres were then oven dried at 50 °C. Autoclaving was not considered to have an adverse effect on the fibres given its short duration. Lignocellulosic fibres have been observed to thermally degrade through dehydration, depolymerisation and oxidation when heated [19], dependent upon temperature and duration of heat exposure. Van de Velde and Baetens [26] reported that after exposing flax fibres to 120 °C for up to 2 h no significant decrease in tensile strength was observed.

#### Pre-Treatment of Jute Fibres

A concentrated cementation medium, CM3, was prepared as per Table 2, to treat fibres to be contained within three of the nine columns prepared. This medium contained the basic chemicals required for the MICP process, urea and calcium chloride in the form of calcium chloride dihydrate, along with Oxoid CM0001 to provide a nutrient source for the bacteria. Oxoid CM0001 (Oxoid Ltd, Basingstoke, UK) is a dehydrated culture medium. The typical 13 g/L solution of Oxoid CM0001 used for the production of liquid broth cultures contains 1 g/L ‘Lab-Lemco’ beef extract, 2 g/L yeast extract, 5.0 g/L peptone and 5.0 g/L sodium chloride.

After following the fibre preparation procedure outlined above, 1 g quantities of jute fibres were placed onto individual 15 cm × 15 cm squares of stainless-steel mesh with draining trays beneath and sprayed with equal amounts of CM3 (approximately 15 mL) until fully covered with this liquid. The fibres were then placed in a sealed plastic container for 24 h to allow for absorption of the CM3, before oven drying at 50 °C for 48 h. Fibres were removed from the mesh and immediately transferred to sealed sterile containers after drying. Due to the hydrophilic nature of the jute, these fibres will readily absorb moisture once exposed to air. The containers were weighed before and after filling (once fibres had cooled to room temperature) to quantify the amount of solid immobilised CM3 on each set of fibres. A set of six treated fibres were prepared due to expected variation in immobilised quantities of CM3, of which three were selected with closely matching quantities of immobilised CM3 for use in this study.

### 2.3. Bacteria Culture

Non-pathogenic (ACDP Group 1) *Sporosarcina pasteurii*, commonly found in soil, was obtained from the American Type Culture Collection, Manassas, VA, USA, (ATCC 11859) as a freeze-dried culture and used to produce a stab culture for storage at 4 °C. Bacteria were transferred from the stab culture using a sterile inoculation loop onto plates of Luria–Bertani (LB) agar amended with 20 g/L syringe filtered urea. Growth medium for plates contained 5 g/L yeast extract, 10 g/L tryptone, 10 g/L sodium chloride, 15 g/L agar and 20 g/L urea in deionised water. The inoculated plates, sealed with gas-permeable film, were incubated at 23 °C room temperature for 48 h. Single colonies from the plates were used to inoculate liquid growth medium. Triplicates of 50 mL liquid broth cultures were produced in 250 mL Erlenmeyer flasks, for use as an inoculant for further liquid broth cultures to be used in experiments. The liquid growth medium consisted of 13 g/L autoclave sterilised Oxoid CM0001 and 20 g/L syringe filtered urea in deionised water. Flasks were shaken at 23 °C, 150 rpm until the late-exponential phase of growth was reached after approximately 12 h and then stored at 4 °C. Liquid broth cultures were produced in 50 mL quantities using 250 mL Erlenmeyer flasks for the batch tests, followed by multiples of 150 mL in 500 mL flasks for the column studies for which greater volumes were required.

Bacterial cultures for use in experiments were inoculated using 100 µL liquid broth culture per 50 mL growth medium and aerobically grown at 23 °C, 150 rpm until an optical density at a wavelength of 600 nm (OD_600_) of 0.9–1.2 was obtained, which equates to approximately 7.5 × 10^7^–1.1 × 10^8^ cells/mL, according to the relationship reported by Ramachandran et al. [27]. For the column studies, freshly grown liquid broth cultures were transferred to 50 mL sterile polypropylene tubes, each containing 35 mL culture, and centrifuged at 5000 rpm for 20 min. The supernatant was then removed, and a sample taken from this to measure the optical density, to take into account any loss of bacteria in the supernatant. The bacteria were then resuspended in a small quantity of phosphate buffered saline (PBS), dispersed using a pipette and transferred to 15 mL centrifuge tubes from which bacteria would be injected in the columns. These bacterial suspensions were made up to 10 mL with additional PBS. Use of PBS ensured the bacteria would not undergo osmotic shock which would otherwise occur in water. Aseptic technique was followed throughout, and involves using lab practices which prevent contamination, to help ensure that the only bacteria present within the culturing flasks and columns was *Sporosarcina pasteurii*.

### 2.4. Preparation of Cemention Medium

Two variations of the cementation medium (CM) were produced for column treatments, as per Table 2. The basic constituents of the CM as required for the process of MICP are urea and a calcium source. Calcium chloride dihydrate was selected for the calcium source. A slightly higher molarity of urea was used in comparison to calcium chloride dihydrate, since this helps ensure all calcium can be utilised. In addition, a source of nutrients, Oxoid CM0001, was added to the cementation medium to promote ongoing bacterial growth and therefore urease activity during treatment. CM1 consisted of 0.67 M urea and 0.50 M calcium chloride dihydrate, in addition to 3 g/L Oxoid CM0001 and was used as a fixation medium to fix the bacteria to the sand within the columns in addition to initiating MICP. CM2, based on the cementation medium used by Stocks-Fischer et al. [8] and Al Qabany and Soga [28], was as per CM1 with 6 g/L Oxoid CM0001, with 0.187 M ammonium chloride and 0.025 M sodium bicarbonate added. Sodium bicarbonate is added to stabilise the pH of the cementation medium before injections [28] and addition of ammonium chloride was found to help stimulate the MICP process beyond the initial CM injection.

The cementation media were prepared using tap water. Results from a batch test conducted as part of this study provided evidence of the beneficial effect of using tap water compared to deionised water. CM1 was produced by first autoclaving a solution containing calcium chloride dihydrate and Oxoid CM0001, into which a solution containing urea was syringe filtered. To prepare 2.0 L of CM2, firstly the ammonium chloride and Oxoid CM0001 were dissolved in 1.6 L tap water. This solution was adjusted to pH 6.0 using 2.0 M HCl prior to then adding the powdered calcium chloride dihydrate. The pH adjustment prevented the calcium precipitating out into the solution. This solution was autoclaved then made up to 2.0 L by adding a solution containing the urea and sodium bicarbonate using a 0.2 µm syringe filter.

### 2.5. Urease Activity and Batch Test

Urease activity (mM urea hydrolysed/min) is calculated as per the relationship derived by Whiffin [7] below, based on a conductivity assay.

(3) $$Urease\;Activity\;=Electrical\;Conductivity\;\left(\frac{\mathrm{mS}}{\mathrm{cm}}/\min\right)\;\times \;11.11\;\left(R_{2}=0.9988\right)$$

Electrical conductivity was measured over five minutes to obtain the average activity per minute, as per Harkes et al. [29]. This process was repeated 3 times for each sample tested and an average taken from the three results.

Specific urease activity (mM urea hydrolysed/min/OD_600_) is further defined by Whiffin [7] as the amount of urease activity per biomass, as per Equation (4).

(4) $$Specific\;Urease\;Activity=\frac{Urease\;Activity}{Biomass\;\left(OD_{600}\right)}$$

A batch test was conducted to determine effects on urease activity of (i) bacterial growth in tap water and deionised water, (ii) inoculation of medium with plate cultures or liquid broth culture, (iii) initial pH of growth medium. 50 mL liquid broth cultures were prepared using tap water or deionised water as described above and grown at 23 °C for 14–19 h, to achieve a stationary stage of growth. The nutrient medium was inoculated with either 100 µL of a liquid broth culture grown to 1.0 OD_600_ or with one colony from a plate culture. To test the effect of pH of the nutrient medium on urease activity the pH of the solution of Oxoid CM0001 nutrient broth in water was adjusted prior to autoclaving and adding urea, after which a 1 mL sample was taken to test the pH prior to inoculation.

### 2.6. Preparation of Columns

**Figure 2:**
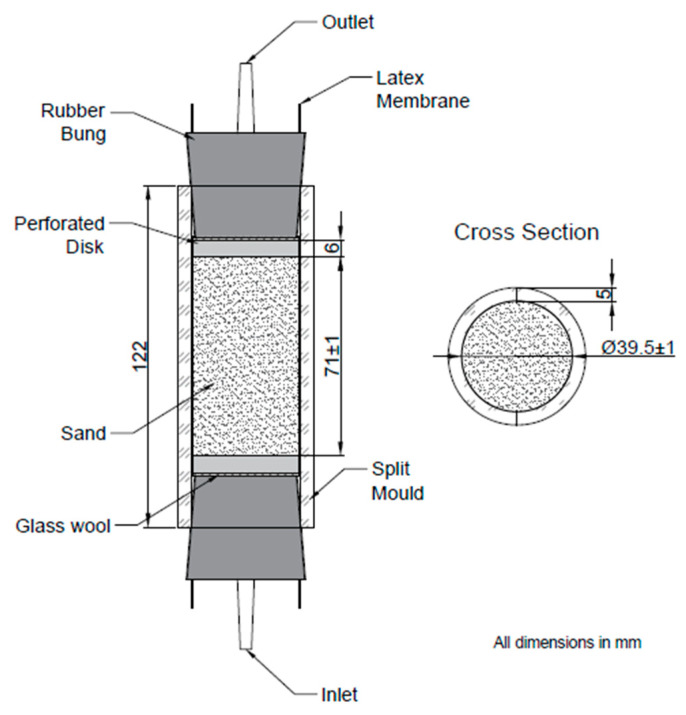
Column assembly.

Columns were assembled as shown in Figure 2. The column apparatus fabricated for this research was based on that used in previous studies by Botusharova [11], with some enhancement including longer split moulds, to ensure that the columns produced would exceed minimum depth to diameter ratios required for the unconfined compressive strength (UCS) testing and to ensure a secure fit of the bungs. Masses of sand and fibres used within columns were as per Table 3. The jute fibre content of columns was 0.75% by weight of sand. Apparatus was washed with 1% Virkon S Disinfectant solution and rinsed with autoclaved deionised water or autoclaved before use to sterilise.

Columns were prepared in triplicates, with J1 to J3 containing sand and fibres, J4 to J6 containing sand and treated fibres and C1 to C3 containing sand only as controls for comparison. Producing the columns in triplicates helps to improve accuracy of results, with results reported as an average from each set of triplicates unless otherwise stated. Each sand column was encased by a 150 mm long, 38 mm diameter, 0.3 mm thick latex membrane, enclosed by clear perspex split moulds of 5 mm thickness and approximately 39 mm inner diameter. To prepare each column, split moulds were secured together using cable ties and the latex sleeve placed inside the mould, with the ends wrapped over the sides of the mould. A perforated 3D printed plastic disk (6 mm depth, 38 mm diameter), wrapped with glass wool was placed into the inlet end of the mould, followed by a bung without the outlet connector in place. The perforated disk was wrapped in glass wool to prevent washout of fine grains of sand during treatments and to provide a gap between the bung and disk. A 3.99 mm × 8 mm silicone tapered plug was inserted into the hole in the inlet bung to retain the CM, into which the sand was deposited using the wet pluviation method combined with vibration. The latex sleeve was wrapped around the inlet bung and this assembly then held upright by a small beaker. A funnel held by a clamp was positioned above the top of the column assembly, leaving 1.5 cm between the funnel tip and column edge. A total of 35 mL of sterile CM1 was poured inside the latex sleeve.

**Figure 3:**
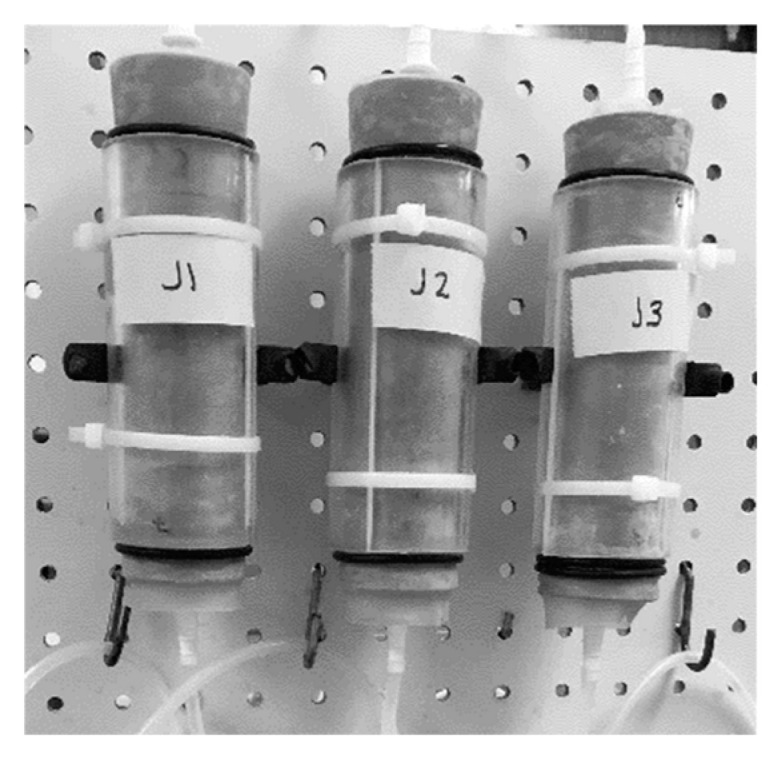
Columns attached to frame.

The column assembly was vibrated three times during the filling process, using a vortex mixer, to aid compaction. The second perforated disk, also wrapped with glass wool, was then placed into the top of the column and the second bung inserted. The bung was pressed firmly into the column while holding the lower bung in place, the membrane was then wrapped around the upper bung and secured using rubber O rings to prevent leaking. This column was then transferred to the column frame, as shown in Figure 3. The column frame consisted of 30 × 60 cm pegboard, with metal feet attached, onto which up to six columns could be secured on either side. This process was repeated for each column.

Prior to addition to the columns, fibres were mixed with sand samples for columns J1 to J6. After weighing the sand and adding fibres a small quantity of CM1 was added, equating to 5% of the total mass of the sand and fibre mixture, to aid mixing. Hydration prior to mixing also helps prevent fibre sand segregation [30]. This was then mixed by hand for approximately five minutes, until an even distribution of fibres was observed. The quantity of liquid added to the sand aided mixing while ensuring that it was still possible to pluviate the sand into the columns. Columns were kept at an ambient room temperature of 23 °C throughout.

### 2.7. Bacteria Fixing and Biocementation

To achieve biocementation, a two-stage process was applied involving (i) the injection of a bacterial suspension, followed by (ii) injections of cementation medium (treatments), as per Table 4. The first treatment, using CM1, is injected immediately after the bacterial suspension and has the effect of fixing the bacteria within the columns in addition to initiating MICP. Harkes et al. [29] found that when a cementation solution consisting of 1 M equimolar urea and calcium chloride was injected into columns immediately after a bacterial suspension this resulted in 100% retention of bacteria, as determined by optical density measurement of effluent samples. Divalent cationic ions such as Ca^2+^ may enhance the attachment of bacteria to surfaces by reducing electrostatic repulsion [31]. The bacterial suspension was injected upwards into the base of all columns simultaneously using a peristaltic pump and constant 1.5 mL/min pumping rate, followed immediately by one and a half pore volumes of CM1. The outlet tubing from columns was then drained and reconnected. Tubing was closed off with clamps and disconnected from the pump tubing following each treatment. On each of the following four days, one and a half pore volumes of CM2 were pumped through the columns, as per the schedule detailed in Table 4.

The timing between treatments had been determined by preliminary studies undertaken as part of this research, and based upon the time taken to deplete the calcium source in columns containing jute, in which this occurred fastest. While injecting CM2 the effluent was collected in a series of 5 mL quantities in 15 mL polypropylene tubes.

### 2.8. Measurement of Electrical Conductivity, pH and Evaluation of Bacterial Fixing

The measurement of pH and electrical conductivity can provide an indication of the extent of substrate conversion, and thus bacterial or urease activity within columns. The effluent displaced from columns during the injection of new CM was collected in a series of centrifuge tubes in 5 mL quantities. For each 5 mL column effluent collected, the conductivity and pH of the effluent was measured using a Consort multi parameter analyser C3010, pH probe and conductivity probe with temperature compensation. Following the first CM treatment, 1 mL of effluent from the 5–10 mL sample of effluent from each tube was taken to measure optical density, as an indication of biomass concentration, to determine effectiveness of bacteria fixing. Optical density was measured using a spectrophotometer (Hach DR 6000, Loveland, CO, USA) at a wavelength of 600 nm. The first 5 mL was not used since this may include some CM that had been retained in the column outlet after treatment.

### 2.9. Geochemical Analysis

The calcium ion concentration of column effluent was measured using an Ion chromatograph (IC). Dionex ICS 5000+ Cation analysis was conducted using 20 mM methanesulfonic acid eluent starting concentration, on a Dionex CS12A column, using 112 mA suppressor output. The first 5 mL effluent from each column was discarded at this stage, since this may contain some unreacted substrates that had been held within the column outlet, outlet tubing had otherwise been drained after each treatment. Beyond this point, up to 20 mL the effluent was mixed for each column to obtain a representative average concentration of calcium ions for each column. The effluent beyond this point was not tested, since this may contain the new CM being injected at later stages in the treatment process as pore volume decreases. Effluent samples were tested daily within two hours of collection to ensure accuracy of results.

### 2.10. Quantification of Calcium Carbonate Precipitate

The calcium carbonate content of the sand columns was quantified using a calcimeter, in accordance with Eijkelkamp [32] and the method of Scheibler, to determine carbonate content based upon a volumetric method. Carbonates in the sample are converted to CO_2_ by adding HCl to the sample. The pressure of the CO_2_ released causes water in a burette to rise and this difference in level from the start of the test enables calcium carbonate content by mass, w(CaCO_3_*)*, to be calculated according to the below equation obtained following calibration, whereby *V*_1_ is the volume of carbon dioxide produced by the reaction of the test portion.

(5) $$w\left(CaCO_{3}\right)=0.0045\;\times V_{1}$$

The result of the above divided by the mass of the sample tested gives the percentage of the sample which contains calcium carbonate. To obtain an estimate of the average calcium carbonate content of each column, as a percentage of the total dry mass, samples of between 4 g and 5 g were taken from the top, centre and base of each column for testing after oven drying at 105 °C. Tests were conducted at constant room temperature of 23 °C.

### 2.11. Mineral Analysis

A combination of scanning electron microscopy (SEM) and X-ray diffraction (XRD) was used to confirm the presence of calcium carbonate and analyse the characteristics of the mineral precipitate in samples taken from biocemented columns. SEM was used to observe the morphology of the mineral crystals and fibres, and to measure the diameter of the jute fibres in the samples. Samples for SEM were prepared using a sputter coater (MCM-100 Ion Sputter Coater, SEC, Korea) and gold coating. X-ray diffraction (XRD; Malvern Panalytical, Aeris powder diffractometer, Malvern, UK) was used to characterise the mineral crystals observed and confirm the presence of CaCO_3_.

### 2.12. Unconfined Compressive Strength Testing

The unconfined compression test was carried out in accordance with 7.2 of BS 1377-7:1990 [33], to determine the unconfined compressive strength (UCS) of biocemented columns using the load frame method. A loading rate of 1.27 mm/min was applied. This test was performed twice, firstly after the biocementation treatment process had been completed (UCS1), and a second time to test for self-healing effects (UCS2). Following the injection of treatment five the cementation medium was left in columns for eight days before UCS1, with the inlet and outlet tubing clamped. The retention time had been longer at this stage due to the significant reduction in calcium ion depletion rate in control columns and also those containing treated fibres. Tested columns were in a saturated condition, with evaporation prevented during testing by the test specimens being encased by the latex membranes and end caps. Membranes were kept in place for this test, perforated discs were removed, and Perspex end caps were used to provide a level testing surface between the column and UCS testing apparatus. Following the initial UCS test, the columns were reconstituted and returned the column assembly, with one and a half pore volumes of autoclaved tap water injected into columns. Samples were left hydrated a further eight days before the UCS test was conducted again. At this stage, the theory that some CM may have been retained, and later leached out of fibres to enable healing was also being tested, following earlier work by Spencer and Sass [10].

## 3. Results and Discussion

### 3.1. Urease Activity Batch Test

Urease activity test results, as per Table 5, show that bacterial growth was faster in a medium prepared using tap water as opposed to deionised water, based on measurements of optical density. Consequently, the cementation medium for the column treatments was prepared using tap water. Liquid broth culture (LBC) inoculant resulted in a higher urease activity than inoculation of the growth medium with a single colony plate culture (PC). It is, however, noted that this may be due to inoculum size, and that this can be better controlled by using liquid broth culture as an inoculant. The liquid broth cultures for the columns were prepared using deionised water (DI). Three measurements of urease activity were taken and averaged for each of the samples prepared.

**Figure 4:**
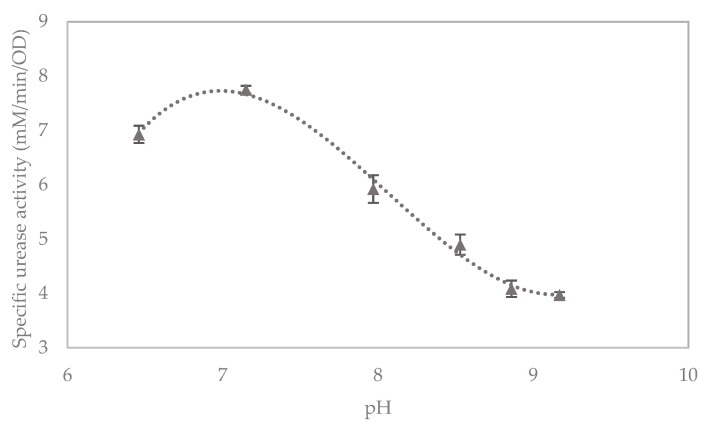
Effect of pH on specific urease activity in liquid broth cultures, with error bars showing standard errors of the means.

The specific urease activity of the liquid broth culture grown using deionised water peaks at a pH of approximately 7, as shown in Figure 4. This is close to the pH of sample 7 above, this being the sample with unadjusted pH, and, hence, the pH of the growth medium was not adjusted for the following column studies.

### 3.2. Column Studies

#### 3.2.1. Soil Properties

**Figure 5:**
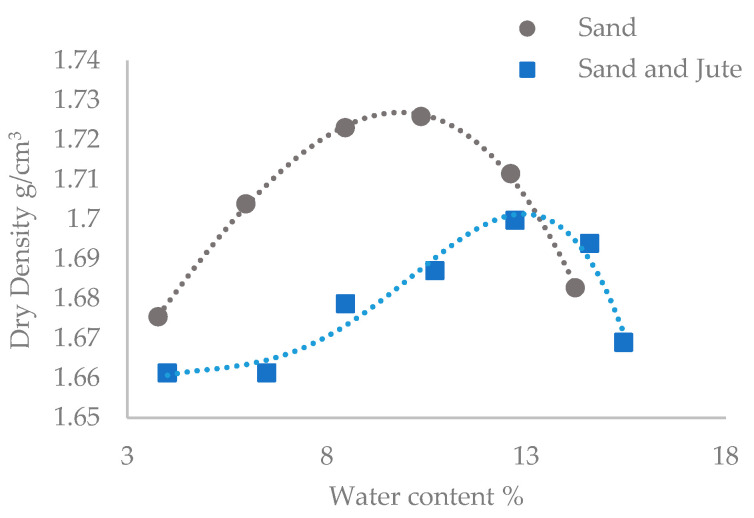
Proctor compaction curves for sand only and fibre and sand mixtures.

Figure 5 gives the optimum dry density (target density) of sand, and the sand and jute mixture, for compaction into the columns, this being 1.726 g/cm^3^ and 1.700 g/cm^3^ respectively. An additional measurement was taken for the test with the sand and jute mixture, as it had been evident during testing that the optimum water content was higher when compared with the sand only test. This had been expected due to absorption of water by the jute fibres. Based on column measurements taken using vernier calipers prior to the first UCS test, columns containing sand only and sand and untreated jute were compacted to 95.7% and 91.4% respectively of their target densities. These results, as an average of the triplicate columns, show that the inclusion of fibres hindered compaction of column contents to some extent.

**Figure 6:**
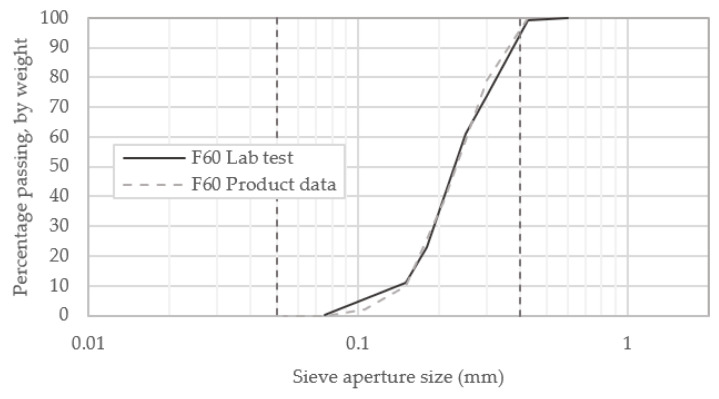
Particle size distribution of F60 sand.

Results obtained for particle size distribution, as shown in Figure 6, are in close alignment with those reported by U.S. Silica. The general slope and shape of this distribution curve are described by means of the coefficient of uniformity (C_u_) and coefficient of curvature (C_z_), with a C_z_ value of between 1 and 3, indicative of a well-graded soil [34]. The parameters determined from the gradation curve for the F60 sand are given in Table 6. The C_z_ value of 1.064 indicates that this soil is fairly well-graded. The low C_u_ value reflects the narrow range of particle sizes.

#### 3.2.2. Bacteria Fixation and Initial Activity

The optical density (OD_600_) of the *S. pasteurii* liquid broth culture grown for the column studies was measured as 0.992 using a spectrophotometer. Following centrifugation, the supernatant optical density was 0.154. Taking into account this loss of bacteria in the supernatant the resulting optical density was 0.840. When using the larger 500 mL Erlenmeyer flasks to produce 150 mL volumes of bacterial cultures, grown for 24 h at 150 rpm and 23 °C, the measured urease activity had been lower than when using the 250 mL flasks to produce the 50 mL cultures. Urease activity of the culture used to inoculate the columns was measured as 4.37 mM/min. The reduced surface area to volume ratio demonstrated the effect of limited oxygen transfer on the urease activity of the culture. The concentration gradient between the oxygen at the surface and within the medium promotes oxygen transfer into the medium [35].

The bacteria appeared to have been successfully fixed by CM1 as shown by the low optical densities of effluent in Table 7, as measured following the injection of CM2. Some bacterial losses were occasionally observed in effluent just after one pore volume of CM had been pumped into the column, when some cloudiness was observed in the effluent. It is noted that should any mineral precipitate be contained within the effluent that this would affect the optical density measurements. However, given the very low optical densities measured, as given in Table 7, this has been deemed to have a negligible effect if any at this stage.

#### 3.2.3. Distribution of Bacterial Activity

**Figure 7:**
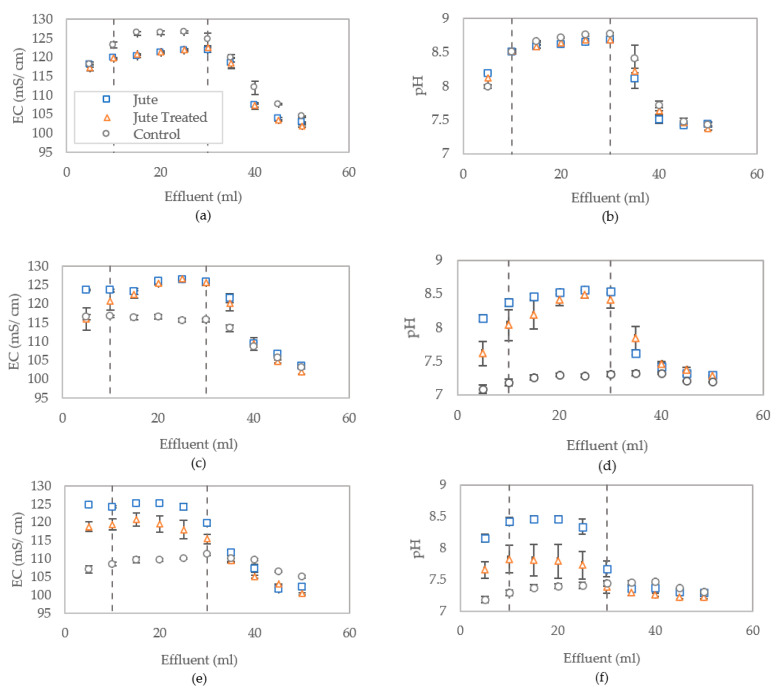

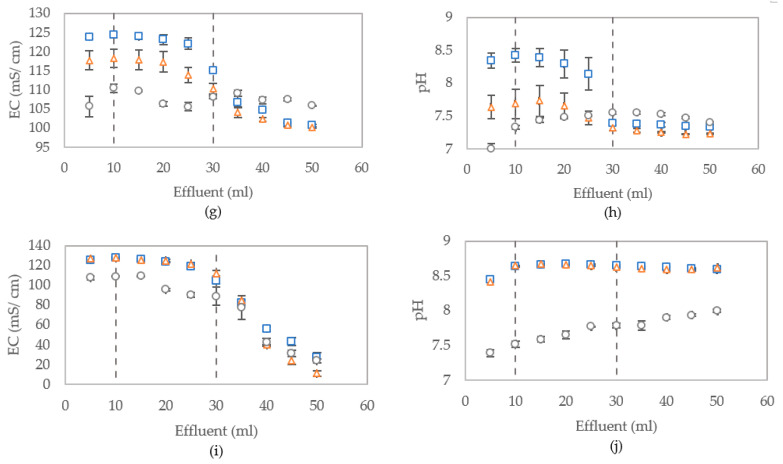
Conductivity and pH of columns effluent measured following treatments 1 (**a**,**b**), 2 (**c**,**d**), 3 (**e**,**f**), 4 (**g**,**h**) and 5 (**i**,**j**), with error bars showing standard errors of the means for the triplicates.

Figure 7a–j shows the breakthrough curves for measured electrical conductivity and pH of effluent displaced during each treatment flush, which are used to analyse the distribution of the reaction products within the columns. These results indicate that the distribution is fairly even for control columns, with more variation in the columns containing jute, as was expected given the jute may absorb some of the bacteria. The results also indicate a slightly lower bacterial activity towards the top of columns (outlet) containing jute following treatment one. This trend was observed to reverse after three CM treatments. The dashed vertical lines in Figure 7a–j represent the interpreted location of column boundaries at the outlet (approx. 5 mL) and inlet (approx. 30 mL) locations.

Conductivity measurements of the effluent from columns show that there is some initial inhibition of MICP activity in columns containing jute fibres (both treated and untreated) during treatment one, as shown in Figure 7a, when compared to the control columns containing sand only, as was similarly observed in aqueous studies reported by Spencer and Sass [10]. However, results from testing of the effluent flushed following subsequent treatments show that for columns containing untreated jute fibres the EC and pH values corresponded to full conversion. The results from effluent tested after treatment three and four show a decline in measured pH and EC from columns containing treated jute compared to the untreated jute. Due to the excess of urea, full conversion of urea would deplete almost all calcium and the remaining solution would be expected to contain about 1.25 mol/L ammonium, 1 mol/L chloride and 0.25 mol/L carbonate/bicarbonate, which according to Van Paassen [36], has an EC of about 125 mS/cm and an expected pH of 8.5 to 9. Incomplete conversion would render lower EC and pH values. The pH and conductivity results in Figure 7 show a similar trend for columns containing treated and untreated fibres, however, the longer error bars for those with treated fibres indicative a greater variability of bacterial activity within these columns.

#### 3.2.4. Efficiency of Calcium Ion Conversion

**Figure 8:**
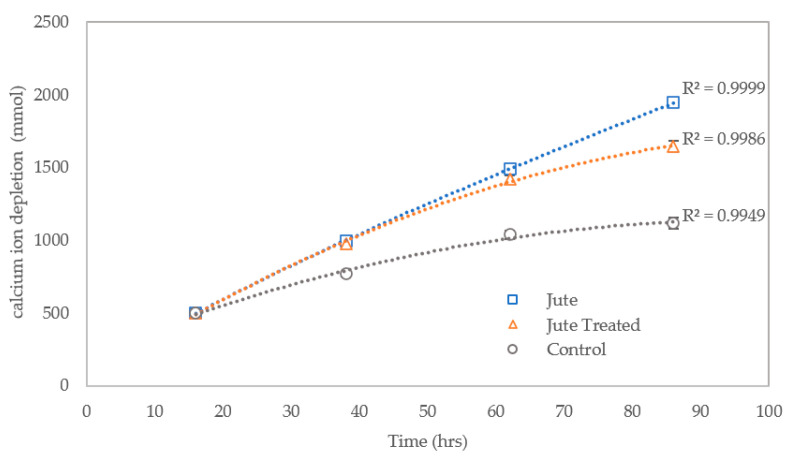
Cumulative reduction in concentration of calcium ions in columns between cementation medium (CM) treatments one and four, with error bars showing standard errors of the means.

The concentration of calcium ions in the effluent has been used as a measure of the efficiency of substrate conversion following CM treatments one to four. The initial calcium ion concentration in the injected CM was 500 mmol, which reduces to an average of 2 mmol across all columns after treatment one. The calcium ion depletion in Figure 8 refers to this reduction in concentration, and has been represented as a cumulative value over time. The concentration of calcium ions in the column effluent shows that the efficiency of conversion of calcium ions to produce calcium carbonate precipitate declines over time for the control columns containing sand only and, to a lesser extent, the columns containing the pretreated jute fibres, between treatments one and four. Where jute has been mixed with the sand the relationship between calcium ion conversion to produce calcium carbonate, i.e., depletion of the calcium ions, in respect of time is almost linear. This clearly demonstrates a beneficial effect of the jute fibres on the MICP process. This effect is likely due to adsorption/absorption of bacteria by the jute fibres, which appears of have had a positive effect on bacterial cell growth/ viability. Cells adsorbed on surfaces replicate and grow into microcolonies [31].

**Figure 9:**
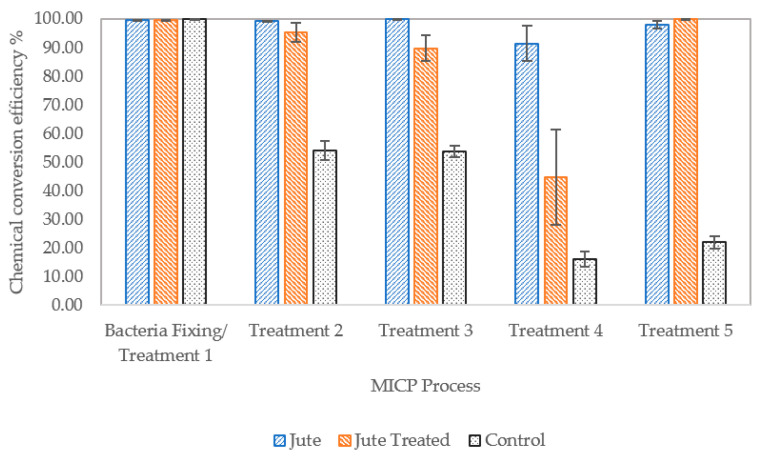
Chemical conversion efficiency, with error bars showing standard error of means.

Figure 9 shows the chemical conversion efficiency following all five CM treatments, based on measurement of calcium ions in the effluent. Following treatment one the conversion efficiency is near 100% for all columns, despite the slightly reduced urease activity of the bacteria injected into columns compared to the batch study. There is a rise in efficiency following treatment five since columns had been left eight days before the first UCS test and subsequent flushing with tap water and collection of effluent. This increase at this stage is significant for the columns containing treated jute and is indicative of a slower but also sustained MICP process when compared to results for columns containing untreated jute. These results, along with those from Figure 7, are indicative of a lower urease activity in columns J4 to J6, suggesting that there are less viable bacteria in these columns at this stage compared to J1 to J3. Based on results in Table 7, bacteria had been fixed adequately following CM treatment one but the fibre pre-treatment may have rendered these fibres less able to absorb/adsorb bacteria and more bacteria may have instead adhered to the sand particles. The adhesion of bacteria to sand particles in columns J4 to J6 is likely somewhere between that of the sand only and sand and untreated jute columns. Adhesion of bacteria to a surface is affected by the physical properties of the surface and surface chemistry, with topography being the most influential factor on bacterial adhesion [31]. The treatment of the fibres may have resulted in a smoothened outer surface, and will likely have also affected their ability to absorb bacteria.

#### 3.2.5. Unconfined Compressive Strength, and CaCO_3_ Precipitated

**Figure 10:**
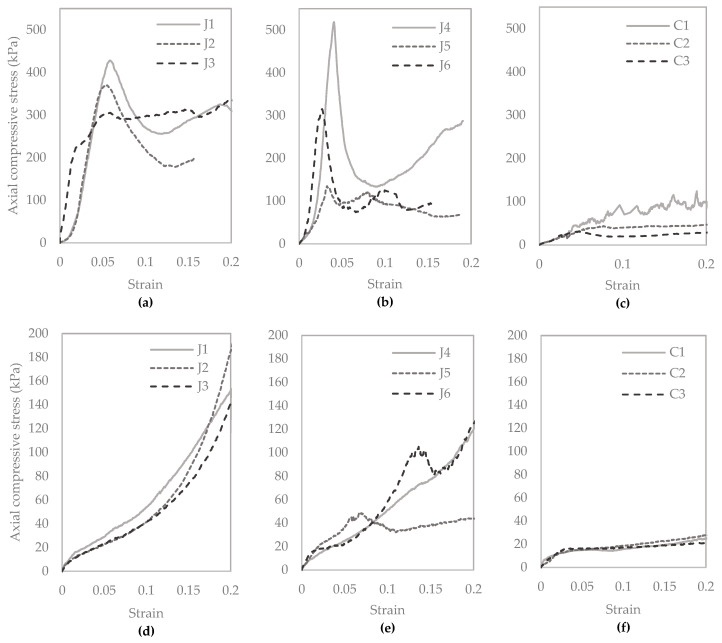
Unconfined compression test results following CM treatment five (**a**–**c**), and after reconstitution, flushing and saturation with water and eight days curing to test for self-healing (**d**–**f**).

**Figure 11:**
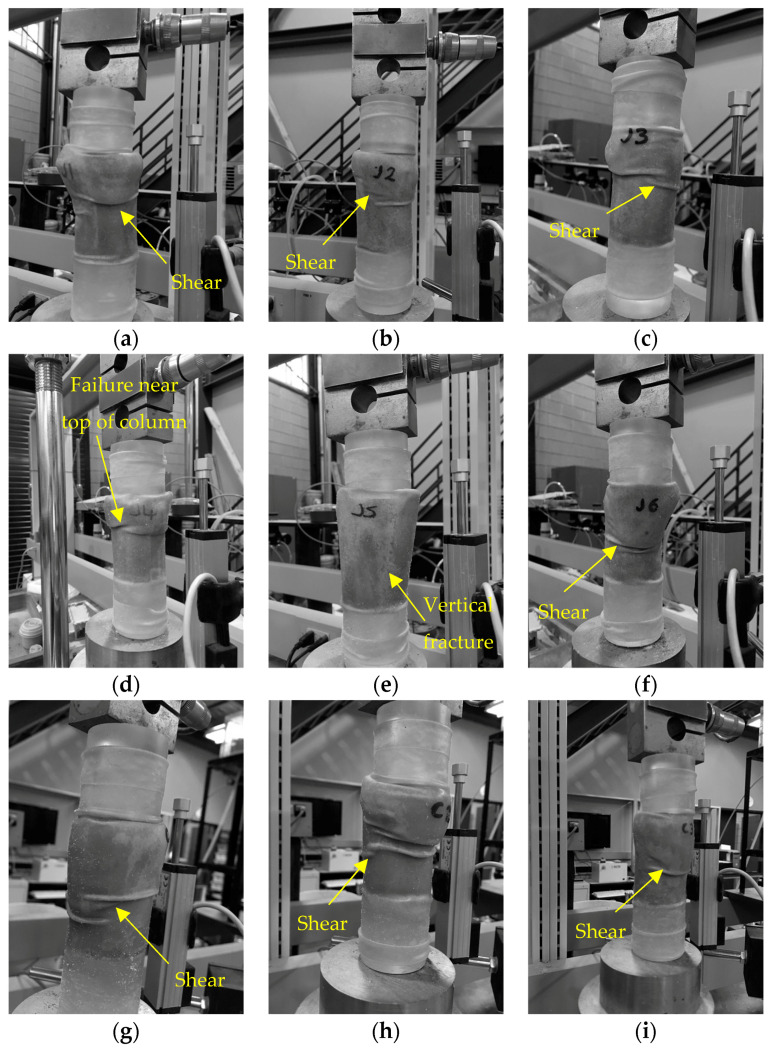
Images of columns J1 to J3 (**a**–**c**), J4 to J6 (**d**–**f**) and C1 to C3 (**g**–**i**), following the onset of failure during unconfined compressive strength testing.

Unconfined compression test results are shown in Figure 10. The average unconfined compressive strength of the three control columns containing sand only was 66 kPa. The average unconfined compressive strength of the columns containing untreated jute and treated jute fibres was 370 kPa, and 320 kPa, respectively. It is observed that on average the unconfined compressive strengths of columns containing untreated fibres are approximately 5.6 times higher than the columns containing sand only. Figure 10a shows a relatively close relationship between the peak unconfined compressive strength results for columns J1 to J3, with the peak strengths all occurring close to 5% strain. This is indicative of good repeatability for these columns. More variation between results is observed in Figure 10b, for columns J4 to J6 containing treated fibres, which appear to fail in a more brittle manner at varying strains between approximately 2.5% and 5%, and have lower residual strengths. For this set of columns, the highest, and also the lowest, UCS is obtained out of the six columns containing fibres. The lower strength for column J5 is attributed to this splitting down the centre during the UCS test, as can be seen in Figure 11e. The highest strength obtained was 520 kPa for column J4 containing treated jute fibres. The variability between results for columns J4 to J6 may have been due to variable absorption or adsorption of bacteria by these fibres. The pre-treatment did not appear to hinder the mixing of fibres with sand.

These results demonstrated the significant contribution to strength of the jute fibres, when compared to controls, as a result of the mechanical properties of the fibres and greater precipitation of calcium carbonate within these columns. The confining effect of the latex membranes will have had a small contribution to strengths obtained, which is assumed to be consistent across all columns tested. Of interest in this study is the comparison between the results for columns tested.

Figure 10d–f is indicative of longer-term strengths of the soil following failure. Between the first set of unconfined compression test results (Figure 10a–c) and second (Figure 10d–f), the column contents contained within the latex membranes were reconstituted, this process itself will have had some effect on material properties and may contribute to some strength reduction. There is a noticeable difference in the trend of the UCS results for the reconstituted samples when comparing Figure 10d–f, with peaks only visible for columns containing treated jute fibres.

Figure 11 shows images of the column samples following the onset of failure during the UCS test. The diagonal shear failure can be clearly seen in most samples. When comparing images, there is a greater inconsistency in observed failure mechanisms for the samples with treated fibres, J4 to J6. J5 was observed to break apart down the centre of the column during testing. The controls typically sheared across the middle third of the test specimen. The columns containing jute had greater resistance to shear failure and the shear failure line appears higher up in the specimen, indicating strengths may have been greater towards the column inlet (base of column).

**Figure 12:**
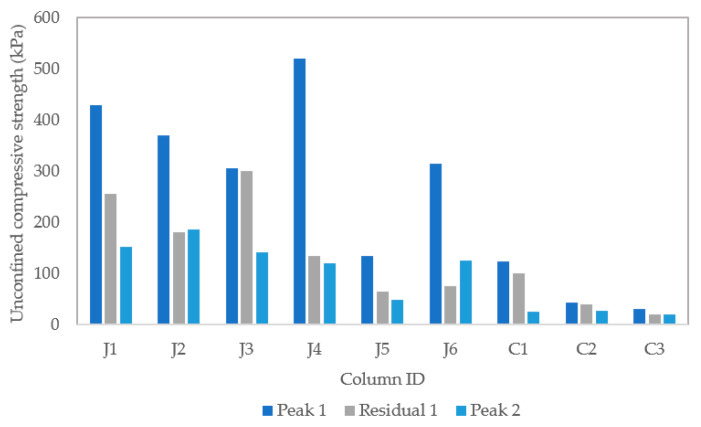
Unconfined compressive strength (UCS) test results.

Figure 12 shows the unconfined compression test results following the initial biocementation (peak 1 and residual 1) and after the reconstitution and self-healing test stage (peak 2). The self-healing stage consisted of saturation with sterile tap water and curing over eight days. When the Peak 2 strength is compared with the residual strength from UCS1, the results for two columns (J2 and most notably J6) indicate some strength regain. In accordance with BS 1377-7:1990 the ‘Peak 2′ unconfined compressive strength has been determined from results at 20% axial strain for columns J1 to J3, J4 and J6 and C1 to C3.

Following the second UCS test, the columns were removed from the latex membranes and oven dried, to determine moisture contents, followed by measurement of calcium carbonate content, as given in Table 8. Columns containing jute will have had a higher void ratio prior to biocementation since columns containing fibres did not compact quite as well those containing sand only. 

The results in Table 8 show that the inclusion of jute and treated jute in the columns had resulted in an increase in calcium carbonate content by 3.69 and 4.33 times on average, respectively, when compared to the columns with no fibres. This increase is significant when compared to studies using synthetic fibres and also shows jute outperforms natural basalt fibres. Choi et al. [13] reported that MICP treated sand specimens containing 0.8% (by weight of sand) PVA fibres had just 1.06 times more calcium carbonate on average than those without fibres. Choi et al. [13] report an average 28.18% unconfined compressive strength increase resulting from PVA fibre additions, although it is noted that there is considerably more variability in the results they obtained. Li et al. [14] found that the UCS of MICP-treated sand with 0.3% (by weight of sand) polypropylene (Fibermesh 150) fibres was 2.4 times higher on average, which reduced to 1.5 times when the fibre percentage increased to 0.4%. Improved results have been achieved using natural basalt fibres. Xiao et al. [15] reported that inclusion of 0.4% basalt fibres in biocemented sand results in a 4.9 times higher unconfined compressive strength on average, and 1.62 times greater calcium carbonate content when compared to specimens with no fibres. Similarly, Xiao et al. [15] reported a UCS reduction to 1.7 times that of sand only specimens when the fibre percentage was doubled to 0.8%.

The greatest amount of calcium carbonate was precipitated within columns J4 to J6, despite the measured reduction in chemical conversion efficiency in these columns following MICP treatments 2 to 4. This is likely due to the leaching of the immobilised cementation medium.

**Figure 13:**
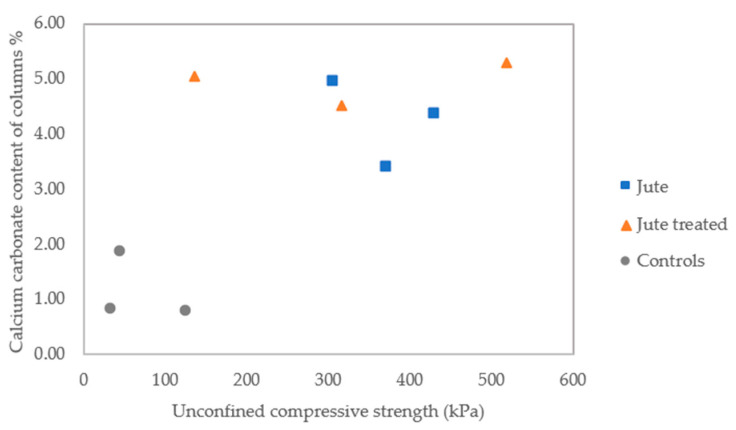
Calcium carbonate content of columns.

The calcium carbonate contents of the nine individual columns, as determined using a calcimeter, are shown in Figure 13. This analysis relates the calcium carbonate contents to the tested unconfined compressive strengths of the columns. There had been a greater consistency between results for the controls and columns containing untreated jute.

#### 3.2.6. Morphology of CaCO_3_ Precipitate and Jute Fibres

**Figure 14:**
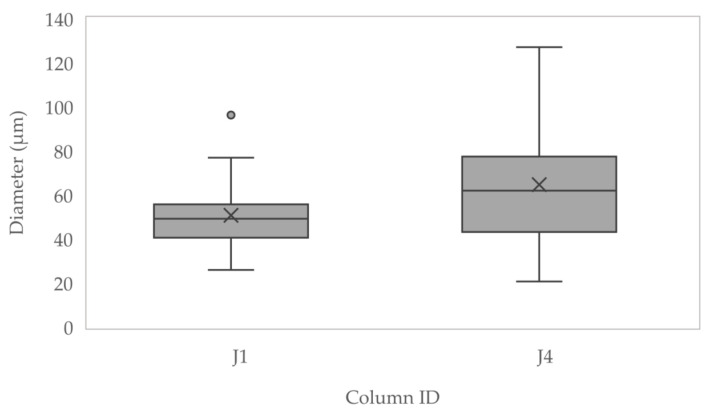
Diameters of jute fibres measured using scanning electron microscopy (SEM).

Samples used for this stage of analysis were J1, J4 and C1. Figure 14 shows the distribution of jute fibre diameters in samples taken from J1 (*n* = 21) and J4 (*n* = 20), measured using SEM. These results indicate the treated fibres had swollen and were more variable in diameter.

**Figure 15:**
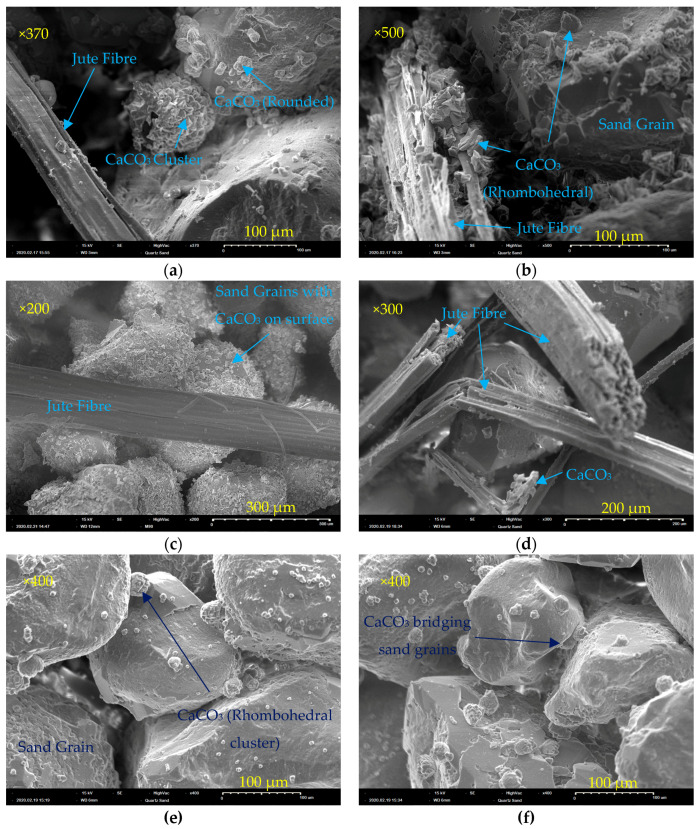
SEM images of samples from biocemented sand columns containing jute (**a**,**b**), treated jute (**c**,**d**), and sand only controls (**e**,**f**).

SEM images of the sample from J1 show that, where there is little to no fibre deterioration observed, as seen in Figure 15a, there is much less CaCO_3_ precipitate observed on the fibre surface when compared to the visibly deteriorated fibre in Figure 15b. The fibre shown in Figure 15b has significant deterioration both on its surface and at depth, since it is breaking apart and has a much more roughened surface covered in CaCO_3_ crystals. Rough fibres will be better at filtering and absorbing bacteria. Fewer crystals were generally observed on the treated jute fibres as can be seen in Figure 15c,d. The fractured fibres shown in Figure 15d suggest perhaps greater brittleness of fibres as a result of the pre-treatment process. The samples containing fibres (Figure 15a–d), show fibres and sand particles with a combination of rhombohedral and rounded crystals of calcium carbonate on the surface. The images of samples containing biocemented sand only, Figure 15e,f, show clusters of what appear to be more rhombohedral shaped calcium carbonate on the sand surface and bridging sand particles. It can be observed that the fibre and sand grain in Figure 15b are bonded together by calcium carbonate crystals and that there were generally a greater number of crystals in jute containing samples. This supports the findings of chemical analyses that show the increased efficiency of substrate conversion to form calcium carbonate in the columns containing jute.

This spherical shape of some of the crystals observed has been associated with crystals of vaterite [28,36,37], with calcite reported to precipitate in a more rhombic form [37]. There are three polymorphs of anhydrous calcium carbonate: vaterite, aragonite and calcite. Calcite is a more thermodynamically stable form of calcium carbonate than vaterite [38]. Experimental evidence has demonstrated that vaterite can transform to aragonite in 60 min at 60 °C and to calcite in 24 h at room temperature [39]. XRD was performed to verify the crystal morphology of the observed precipitate.

**Figure 16:**
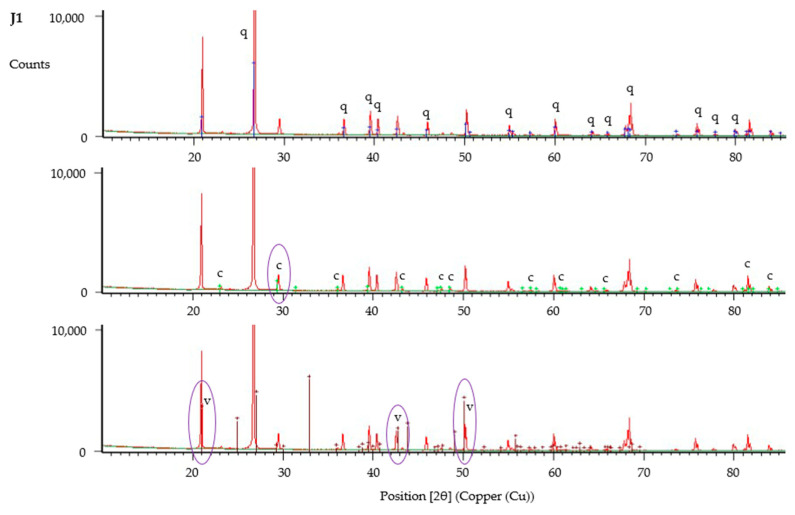
X-ray powder diffraction (XRD) analysis of sample from column J1, showing identified peaks of quartz (q), calcite (c) and vaterite (v).

**Figure 17:**
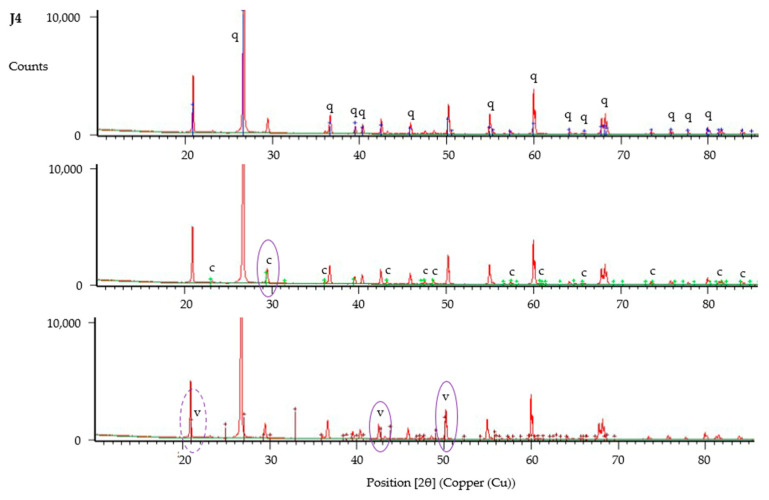
XRD analysis of sample from column J4, showing identified peaks of quartz (q), calcite (c) and vaterite (v). The peak at 21 [°2θ] is identified as vaterite based on results for J1 and C1, however results for J4 alone suggest this could be also be quartz.

**Figure 18:**
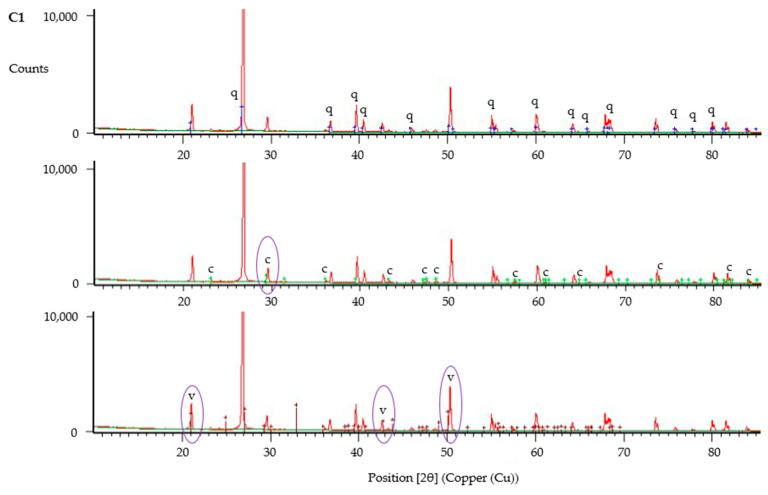
XRD analysis of sample from column C1, showing identified peaks of quartz (q), calcite (c) and vaterite (v).

The XRD data was analysed using HighScore Plus, with results shown in Figure 16, Figure 17 and Figure 18. XRD data have been compared with reference patterns to determine crystalline phases present, with phases identified based upon the closest match between intensity and position of reference patterns and the diffraction peaks. These analyses verify the presence of calcite and vaterite polymorphs of calcium carbonate in all samples tested. Significant peaks for each crystalline phase are shown circled in Figure 16, Figure 17 and Figure 18. These results indicate that vaterite may be the more dominant of the calcium carbonate polymorphs present within all samples, in particular those with untreated fibres (J1–J3), based upon height of peaks and intensity of the reference pattern. Nawarathna et al. [40] reported that addition of chitosan as an organic additive to enhance MICP promoted the production of vaterite. This suggests that jute as an organic material may be influencing the crystal morphology in a similar manner, leading to the observed dominance of vaterite, due to the physicochemical properties of these fibres.

## 4. Conclusions

Column studies were undertaken to investigate the effect of jute fibres on both the process of MICP and on properties of biocemented sand. Biocemented sand columns were produced in triplicates, containing sand and untreated jute fibres, sand and treated jute fibres and sand only as controls. The treated fibres immobilised a concentrated cementation medium, with the aim of enabling self-healing via MICP.

The results showed that the incorporation of jute fibres within a biocemented sand material significantly increased the unconfined compressive strength of this material when compared to biocemented sand without the jute fibres. This strength increase results from the contribution to strength properties of not just the fibres themselves, but also the increased amount of calcium carbonate precipitated in the columns containing jute fibres. On the basis of the results obtained the contribution to unconfined compressive strength increase by the fibres alone cannot be ascertained. In addition to increasing strength, the inclusion of fibres had a beneficial effect on the MICP process, improving efficiency of substrate conversion, likely as a result of sustaining the bacterial growth and, hence, urease activity. It is likely that bacteria had been absorbed by the jute and also adsorbed onto the surface of the fibres within the columns, and that this contributed to the positive findings. More investigation would be required to fully understand this effect. Tuson et al. [41] reported that bacterial systems used for sensing and responding to surfaces are still not well understood. Surface roughness of the fibres also appears to have added to this effect given the observed higher density of calcium carbonate crystals observed on the surface of roughened fibres. Renner et al. reported the significant influence of surface topography on bacterial adhesion [31].

A consequence of the fibre inclusions within the biocemented sand sustaining longer term activity of *Sporosarcina pasteurii* bacteria is the enabling of the continuation of the MICP process without the need for multiple injections of bacteria. This could reduce the cost of production of a biocemented sand material and would be beneficial where several treatments of cementation medium are required to achieve a low permeability and/or high strength.

The evaluation of self-healing effects and quantification of this has proved challenging, with only one column containing treated fibres showing any significant potential self-healing capacity. To achieve self-healing via MICP the cementation medium would need to be stored within the biocemented soil matrix for later release. Immobilisation may only be effective if a material embedded within the biocemented soil can retain sufficient cementation medium during the initial MICP treatment process. Therefore, a material which enables a sufficiently slow release of immobilised chemicals would be required. More testing is required on this aspect and alternatives such as encapsulation explored. A self-healing MICP system may be of particular interest and suitability for seepage control, such as within a dam core or within grouting. For this application, a sufficient amount of cementation medium treatments would need to be applied to achieve a very low permeability, which the addition of jute fibres may help to facilitate. It is then assumed the self-healing MICP process would be activated upon water ingress into this material should micro-cracking occur, to help prevent piping for example.

The results from the set of columns containing the treated fibres may give some insight into effects of pre-treating fibres prior to use in MICP applications. These fibres were subject to chemical treatment with the concentrated cementation medium, and also some additional heat treatment, while these fibres were dried at 50 °C. This is an area which could be explored further and had not been a focus of this study. This study could be further extended using recycled jute fibres. It is expected that surface roughness of recycled fibres may further promote fixing of bacteria to fibres, however, any processing treatment fibres have undergone, contamination of fibres and potential deterioration should also be taken into consideration.

## Figures and Tables

**Table 1:** Sand properties.

Soil Origin	G_s_	ρ (g/cm^3^)	Mineralogy	Shape
Ottawa	2.65	1.522	Quartz	Round

**Table 2:** Cementation media composition and sterilisation methods.

Precursor Chemicals and Nutrients	CM1 (g/L)	CM2 (g/L)	CM3 (g/L)	Sterilisation Method
Calcium chloride dihydrate (CaCl_2_∙2H_2_O)	73.51	73.51	147.02	Autoclaved
Urea (NH_2_(CO)NH_2_)	40	40	80	Syringe filtered
Ammonium chloride (NH_4_Cl)	0	20	-	Autoclaved
Sodium bicarbonate (NaHCO_3_)	0	2.12	-	Syringe filtered
Oxoid CM0001 nutrient broth	3	6	12	Autoclaved

**Table 3:** Column contents.

Column ID	Sand, g	Jute, g	Immobilised CM, g
J1	133	1	0
J2	133	1	0
J3	133	1	0
J4	133	1	1.687
J5	133	1	1.681
J6	133	1	1.686
C1	143	0	0
C2	143	0	0
C3	143	0	0

**Table 4:** Columns treatment schedule.

Day	Time Since Prior Injection (h)	Column Injection (1.5 × Pore Volume)	Treatment
0	0	CM1	1
1	16	CM2	2
2	22	CM2	3
3	24	CM2	4
4	24	CM2	5
12	192	Tap Water	None

**Table 5:** Results from urease activity tests.

ID	Water	Inoculant	pH	Culture Time	OD	Electrical Conductivity (mS/cm/min)	Urea Hydrolysed (mM/min)	Specific Urease Activity (mM/min/OD)
1	Tap	PC	8.37	19	0.940	0.56	6.22	6.62
2	DI	PC	7.97	19	0.937	0.41	4.59	4.90
3	Tap	LBC	8.37	19	1.308	0.75	8.30	6.34
4	Tap	LBC	8.37	16	1.222	0.69	7.63	6.24
5	Tap	LBC	8.37	14	1.068	0.57	6.30	5.89
6	DI	LBC	6.46	19	1.005	0.63	6.96	6.93
7	DI	LBC	7.15	19	0.822	0.57	6.37	7.75
8	DI	LBC	7.97	19	1.038	0.55	6.15	5.92
9	DI	LBC	8.53	19	1.134	0.50	5.56	4.90
10	DI	LBC	8.86	19	1.196	0.44	4.89	4.09
11	DI	LBC	9.17	19	1.156	0.41	4.59	3.97

**Table 6:** Sand parameters.

D_10_	D_50_	D_60_	D_30_	C_u_	C_z_
0.140	0.226	0.250	0.193	1.786	1.064

**Table 7:** Optical density of column effluent following bacteria fixing.

Column	J1	J2	J3	J4	J5	J6	C1	C2	C3
Effluent OD_600_ (5–10 mL)	0.018	0.01	0.015	0.011	0.008	0.011	0.055	0.02	0.044

**Table 8:** Moisture and CaCO_3_ contents of biocemented columns (averages from triplicates).

Columns	Moisture Content %	CaCO_3_ Content%
J1–J3	18.35 ± 0.34	3.98 ± 0.59
J4–J6	16.82 ± 1.33	4.63 ± 0.31
C1–C3	17.03 ± 0.39	1.08 ± 0.47

## Abbreviations

The following symbols are used in this paper.

Ρ	Bulk density
G_s_	Specific Gravity
D_10_	Diameter corresponding to 10% finer in the particle-size distribution
D_50_	Diameter corresponding to 50% finer in the particle-size distribution
D_60_	Diameter corresponding to 60% finer in the particle-size distribution
D_30_	Diameter corresponding to 30% finer in the particle-size distribution
C_u_	Uniformity Coefficient of uniformity
C_z_	Coefficient of curvature
